# Desperate Times Call for Desperate Measures: Use of Continuous Subcutaneous 1-34 PTH Infusion for Postsurgical Hypoparathyroidism

**DOI:** 10.1155/2021/5593653

**Published:** 2021-03-09

**Authors:** Adnan Haider, Oksana Symczyk, Yadel Couso, Taylor Cater, Sheree Bryan

**Affiliations:** ^1^Department of Endocrinology, West Virginia University, Morgantown, WV, USA; ^2^Department of Medicine, West Virginia University, Morgantown, WV, USA

## Abstract

**Objective:**

This case highlights use of 1-34 PTH continuous infusion in a patient with postsurgical hypoparathyroidism.

**Method:**

Clinical presentation and biochemical profile were monitored before and after 1-34 PTH infusion, with notable reduction in pill burden in a patient with postsurgical hypoparathyroidism.

**Results:**

We present a case of postsurgical hypoparathyroidism following thyroidectomy for Graves disease. The patient was requiring a total of 34 pills daily and, despite medication compliance, her clinical and biochemical control was unsatisfactory. Following initiation of 1-34 PTH in the form of a subcutaneous pump, we were able to stop all calcium supplementation and reduce calcitriol to 0.5 mcg daily. Her current biochemical control as well as quality of life improved significantly on CSPI, calcitriol, and a daily serving of dietary calcium.

**Conclusion:**

This case highlights the use of 1-34 PTH either as twice-daily dosing or continuous subcutaneous infusion for adult patients with hypoparathyroidism.

## 1. Introduction

Hypoparathyroidism is a rare endocrine disorder, in contrast to its pathological counterpart primary hyperparathyroidism. In the US and European Union, hypoparathyroidism is classified as an orphan disease. Hypoparathyroidism is the last classical endocrine deficiency for which the missing hormone has become available. Before the use of recombinant PTH in clinical practice, management of hypoparathyroidism was limited to calcium and active vitamin D, often in large doses and with the adverse effect of extraskeletal calcifications. However, without PTH, normal calcium homeostasis, via skeleton, kidney, or central nervous system mechanisms, cannot be restored [1, 2].

It is virtually impossible to manage hypoparathyroidism by providing sufficient calcium via diet alone. Calcium supplements and active vitamin D products are essential. Typically, patients will require 1–2 g of supplemental calcium given in divided doses. Some patients will require higher doses. The FDA approved rhPTH (1-84) for patients with hypoparathyroidism who cannot be controlled on conventional therapy in January 2015, a signal event in the therapeutic history of hypoparathyroidism. 1-84 was especially useful for patients requiring higher doses of oral calcium supplementation (>2.5 g/day or active vitamin D > 1.5 mcg/day). Another indication for 1-84 use is poor control of serum calcium (corrected serum calcium < 7.5 mg/dl or clinical symptomatology of low calcium despite high calcium intake). However, in September 2019, rhPTH (1-84) was recalled due to a potential issue related to particulates originating from the rubber septum of the NATPARA cartridge.

## 2. Case Presentation

A 34-year-old white female diagnosed with Graves hyperthyroidism was initially treated for about 18 weeks with methimazole without improvement in thyroid levels because of severe endometriosis history, bilateral oophorectomy, and hysterectomy five years before her Graves diagnosis. Her gynecologist did not start hormone replacement therapy on account of her smoking history. Graves orbitopathy and smoking history made RAI ablation contraindicated. She opted for total thyroidectomy to treat her large goiter and associated pressure symptoms. She developed immediate postoperative hypoparathyroidism, presenting with symptomatic hypocalcemia within 24 hours of total thyroidectomy. Pathology reports of the surgical specimen demonstrated diffusely enlarged thyroid along with two parathyroid glands. During hospitalization, she was treated with intravenous calcium gluconate drip and was discharged home on calcium carbonate, vitamin D, and active vitamin D.

Despite her adherence to the discharge regimen, she experienced hypocalcemia symptoms of fatigue and frequent cramping in the hands. She required regular ER visits for symptomatic hypocalcemia. A PICC line was placed due to frequent hospitalizations and the need for calcium monitoring every two weeks. In the six months following her surgery, she required eight ER visits with four overnight stays in the hospital. During hospitalizations, the patient was treated with intravenous calcium gluconate and was discharged on oral calcium and active vitamin D regimen. Postsurgical hypothyroidism remained under reasonable control on a stable levothyroxine dose.

The patient denied the previous history of malabsorption, and celiac disease screening was negative as well. Elevated 24-hour urine calcium before initiation of hydrochlorothiazide was 402 mg/24-hour urine specimen.

The patient's thyroidectomy was performed in August 2019. In September 2019, that 1-84 PTH was recalled and not available for 1-84 PTH naïve patients. At the time of initial consultation in this endocrinology clinic (May 2020), the patient's medications included more than 30 pills daily (Table 1).

The initial plan is summarized in Table 2. Due to the current unavailability of 1-84 PTH (Natpara), it was impossible to start her on Natpara. Because of patient's symptomatic hypocalcemia and frequent hospitalizations despite taking her pills, a decision was made of start 1-34 PTH (Teriparatide) 20 mcg initially as twice-daily subcutaneous injections. Because of the short half-life of 1-34 PTH (Teriparatide) in previous studies, we prescribed twice-daily injections for her.

**Figure 1:**
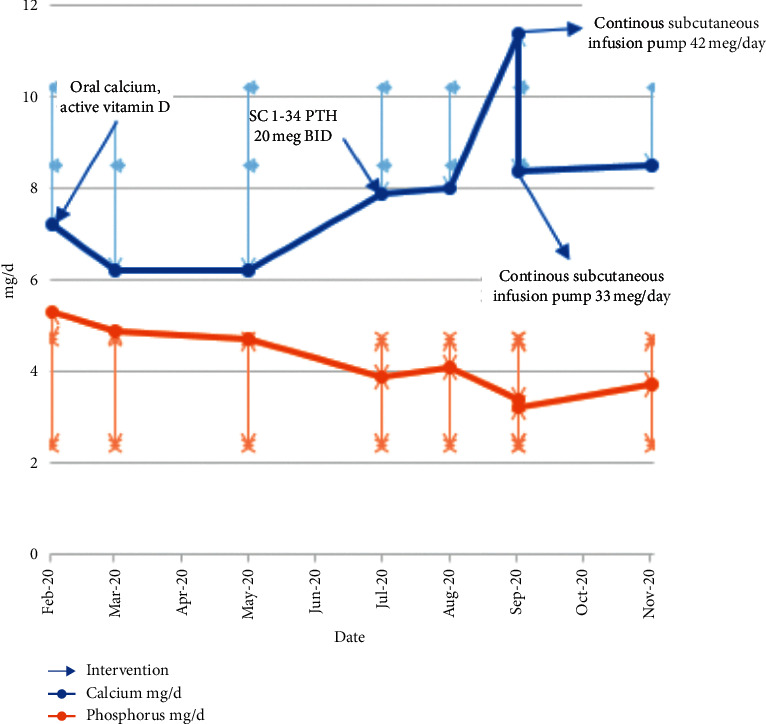
Review of her phosphorus and calcium levels prior to oral calcium and vitamin D replacement, to subcutaneous 1-34 PTH therapy, and to CSPI summarized.

The patient's calcium dose was reduced by half at the initiation of twice-daily 1-34 dosing, and her calcium levels improved (Figure 1). Her calcium levels improved from 6.4 mg/dl to 7.9 mg/dl on 1-34 PTH, with symptomatic improvement and 50% reduction in pill burden. To further improve her calcium hemostasis, by simulating the physiologic tonic release of PTH, a decision was made to give the teriparatide via a continuous subcutaneous pump. Initially, a dose of 42 mcg/day was infused via an Omnipod pump. At this dose, patient calcium increased from 7.7 mg/dl to 11.2 mg/dl, at which point all calcium supplements and active vitamin D were stopped, and a dose of teriparatide was reduced to 33 mcg/day. Follow-up blood work showed normal calcium and phosphorus levels. With the use of continuous subcutaneous infusion, we observed a 17.5% dose reduction in daily 1-34 PTH and improvement in calcium and phosphorus levels, as shown in Figure 1.

## 3. Discussion

Can 1-34 PTH (Teriparatide) provide relief to patients with hypoparathyroidism until 1-84 PTH (NATPARA) is available again?

Will continuous subcutaneous parathyroid infusion of teriparatide provide any advantage in the long run when compared to single daily 1-84 PTH?

A single daily injection of rhPTH improved quality of life, reduced pill burden, and improved calcium levels and hypocalcemia symptomatology in patients with hypoparathyroidism. This agent was especially useful in patients with gastrointestinal dysfunction or following bariatric surgery where unpredictable calcium absorption can make disease management especially challenging. Despite its advantages, rhPTH (1-84) therapy for any length of time can lead to dangerous hypocalcemia once therapy is discontinued: rhPTH (1-84) activates the skeletal system and, as a result, the skeletal bone will continue to accrue calcium, but if treatment is interrupted, hypocalcemia can result [3]. Patients on rhPTH (1-84) are instructed to increase their calcium and active vitamin D to levels that match or surpass the amounts they were taking before starting rhPTH (I-84) therapy.

In the late 1980s, Winer and colleagues demonstrated that 1-34 PTH, an active fragment of PTH, could be used effectively in children and adults with hypoparathyroidism [4]. The short half-life of 1-34 PTH is what makes twice- or thrice-daily dosing more effective in controlling calcium levels. 1-34 PTH improved control of serum calcium and reduced supplementation with calcium and active vitamin D [5, 6].

Andre Palermo studied the effects of 1-34 PTH 20 mcg twice-daily subcutaneous injections in a prospective open-label study. This study showed improvement in calcium levels and reductions in calcium and vitamin D supplementation. No serious adverse events occurred during the study period, no subjects developed nephrolithiasis, and no hypercalcemic events necessitated hospitalization, and over the two years, these patients were using subcutaneous 1-34 PTH injections [6].

Continuous infusion of PTH would mimic the tonic secretory dynamics that represent the majority of secreted PTH under normal circumstances and thus provide advantage over multiple daily injections of 1-34 PTH. Continuous infusion of 1-34 PTH resulted in 65% less rhPTH required to control the serum calcium when compared to a twice-daily injection regimen. Review of the literature indicates that continuous PTH infusion can mimic the tonic release of PTH and can lead to not only better calcium homeostasis but also to reduced total daily dose of 1-34 PTH. Additionally, continuous infusion of 1-34 PTH reduces lower urinary calcium excretion by 50% [7].

Continuous subcutaneous parathyroid infusion (CSPI) allows long-term maintenance of serum and urinary calcium at near-normal values as was demonstrated by Agnes Linglart in three children with hypoparathyroidism [8]. Of significance, CSPI corrected the clinically severe manifestation of hypocalcemia that impeded the children's lives daily.

In rodent studies, all PTH and PTH-related protein molecules, when tested at high doses for prolonged periods, were associated with osteosarcoma [9, 10]. All PTH molecules approved for human use carry with them the black box warning of osteosarcoma. Although the FDA has approved rhPTH (1-84) for long-term use in hypoparathyroidism patients without risk factors for osteosarcoma, restrictions as to the duration of use have not been dictated and so the black box warning persists. Continuous subcutaneous 1-34 PTH for long-term use is valid a concern. However, after nonhuman primate studies and more than 17 years of human surveillance, no safety signals are evident [11, 12]. Furthermore, osteosarcoma is not a safety signal in human subjects.

Desperate times call for desperate measures. Use of 1-34 PTH, two to three injections daily, or CSPI provides an alternative to 1-84 PTH for patients who are difficult to manage with currently available treatment such as our case patient. Use of CSPI mimics the tonic secretory dynamics that represent the majority of secreted PTH under normal circumstances, a potential advantage in treating hypoparathyroidism.

## 4. Conclusion

This case highlights the use of 1-34 PTH for postsurgical hypoparathyroidism. Significant biochemical and clinical improvement was achieved. Significant reduction in pill burden was also reduced following initiation on 1-34 PTH injections.

Using CSPI, the total daily dose reduction of 1-34 PTH was observed when compared to twice-daily injections and, at the same time, better biochemical control was noted.

Can 1-34 PTH provide relief to patients with hypoparathyroidism until 1-84 PTH (NATPARA) is available again? The outcome of this case provides hope.

Will CSPI infusion of 1-34 PTH analogue provide any advantage in the long run when compared to single daily 1-84 PTH? A randomized double-blind crossover trial will be the best suited study to answer the two questions and better understand the pathophysiology of hypoparathyroidism.

## Figures and Tables

**Table 1:** List of medications.

List of medications
1. Calcium citrate 500 mg, 2 tablets, four times daily
2. Calcium carbonate 750 mg, 3 tablets, four times daily
3. Calcitriol 0.5 mg, four times daily
4. Calcium acetate 667 mg, 2 tablets. 3 times daily
5. Hydrochlorothiazide 50 mg daily
6. Magnesium citrate 300 mg daily
7. Ergocalciferol 50, 000 IU weekly
8. Levothyroxine 200 mcg daily

**Table 2:** Plan during initial visit and outcomes.

Plan during initial visit and outcomes
1. Smoking cessation
Stopped smoking 3 weeks after initial consultation
2. Transdermal estradiol patch for postsurgical menopause
Started 2 weeks after smoking cessation
3. I-34 PTH, 20 mcg, twice daily approved by insurance
50% reduction in calcium supplements and active vitamin D
4. Omnipod continuous subcutaneous pump approved
Initial dose: I-34 PTH infusion 42 mcg/24-hour period via Omnipod pump
Dose adjustment: I-34 PTH infusion 33 mcg/24 hour daily plus calcitriol 0.5 mcg daily All calcium supplements and hydrochlorothiazide were stopped at this time
